# 3D-Printed Solid Dispersion Drug Products

**DOI:** 10.3390/pharmaceutics11120672

**Published:** 2019-12-11

**Authors:** Suet Li Chew, Laura Modica de Mohac, Bahijja Tolulope Raimi-Abraham

**Affiliations:** 1 Drug Delivery Group, Institute of Pharmaceutical Science, Faculty of Life Sciences and Medicine, King’s College London, Franklin-Wilkins Building, 150 Stamford Street, London SE1 9NH, UK; 2 Department of Sciences for Health Promotion and Mother-Child Care “G. D’Alessandro”, University of Palermo, 90100 Palermo, Italy

**Keywords:** 3D printing, amorphous solid dispersion, additive manufacturing, poor solubility, fixed dose combination

## Abstract

With the well-known advantages of additive manufacturing methods such as three-dimensional (3D) printing in drug delivery, it is disappointing that only one product has been successful in achieving regulatory approval in the past few years. Further research and development is required in this area to introduce more 3D printed products into the market. Our study investigates the potential of fixed dose combination solid dispersion drug products generated via 3D printing. Two model drugs—fluorescein sodium (FS) and 5-aminosalicyclic acid (5-ASA)—were impregnated onto a polyvinyl alcohol (PVA) filament, and the influence of solvent choice in optimal drug loading as well as other influences such as the physicochemical and mechanical properties of the resultant filaments were investigated prior to development of the resultant drug products. Key outcomes of this work included the improvement of filament drug loading by one- to threefold due to solvent choice on the basis of its polarity and the generation of a 3D-printed product confirmed to be a solid dispersion fixed dose combination with the two model drugs exhibiting favourable in vitro dissolution characteristics.

## 1. Introduction

A fixed-dose combination (FDC) product is a single dosage form that incorporates two or more active pharmaceutical ingredients (APIs) [1,2]. Between 2013 and 2018, the European Medicines Agency (EMA) approved 66 FDCs, most of which were antiretrovirals for human immunodeficiency virus (HIV) infections [3]. FDC products have several advantages over conventional medicinal products, namely, greater efficacy (43%, *n* = 33) and compliance (18%, *n* = 14) [4,5,6,7]. However, disadvantages have been highlighted, such as a reduction in medication adherence (24%–26%) in some cases [8,9].

The use of three-dimensional (3D) printing in drug delivery is still in its infancy compared to traditional technologies; however, research and development is rapidly expanding in this area due to the benefits of 3D printing to develop personalized patient-specific dosage forms with tailored release profiles [10,11,12,13]. Traditional powder direct compression techniques to generate FDC medicinal products is not suitable [14,15,16,17,18,19]. Currently, the only regulatory approved (by the Food and Drug Administration (FDA)) 3D printed medicinal product is the oro-dispersible levetiracetam tablet, Spritam developed by Aprecia Pharmaceuticals in 2015 [20]. The number of regulatory approved 3D printed drug products remains limited due to the number of printers available to comply with good manufacture practice (GMP), high variability of 3D printers, and end product quality [21,22,23,24]. Fused deposition modelling (FDM) uses heat to melt thermoplastic polymers into the molten state and the object to be printed is designed by computer-aided drafting, which enables it to be printed layer-by-layer as the printer nozzle deposits the extrudate [15,25]. FDM 3D printing has been explored extensively in the development of medicinal products and, more specifically, FDC products. FDM 3D printing is capable of producing drug products with multiple active pharmaceutical ingredients in various compartments, which is advantageous in developing patient-centric formulations to reduce multiple daily dosing, therefore improving patient compliance and therapeutic efficiency [26,27,28]. 

The use of solid dispersion technology has been explored in FDM 3D printing [26]. In the study described here, we firstly explored the influence of solvent type on filament (polyvinyl alcohol (PVA)) drug loading using the drug impregnation method. We then manufactured solid dispersion FDC 3D printed dosage forms using the drug-solvent-filament combination, which gave the highest drug loading. Physicochemical characterization of the filaments was conducted and an evaluation of filament and FDC mechanical properties by way of hardness and tensile strength were also evaluated. In vitro drug dissolution studies on the FDC 3D printed dosage forms were also conducted [29,30]. 

Several studies have used the drug impregnation method to load drugs onto polymer filaments for 3D printing. In the case of PVA filaments, this is commonly done by soaking the filament in a highly saturated drug solution. However, this method can result in low drug-loading (<2% *w*/*w*) due to slow drug diffusion into polymer [17,24,29,30,31]. The general drug-loading differences using different solvents such as ethanol (EtOH) and methanol (MeOH) for this filament drug loading method still remains relatively unknown. Studies conducted on 3D-printed FDC often separate APIs into different compartments such as the DuoCaplet design by Goyanes et al. [23]. The potential of FDM-printed monolithic FDC design, by incorporating two APIs into the same polymer filament, is yet to be investigated in terms of ability to independently tailor the different APIs release. This study aimed to explore the potential of increasing drug-loading efficiency by altering solvent choice, and to study the in vitro dissolution profiles of the FDM-printed monolithic FDC tablet developed. Fluorescein sodium (FS) and 5-aminosalicyclic acid (5-ASA) were chosen as model drugs due to their proven FDM-printability [29,30]. PVA was selected as main polymer as it is the only commercially extruded polymer filament that would dissolve in vivo [29].

## 2. Materials and Methods

### 2.1. Materials

Fluorescein sodium salt (FS, 376.27 g/mol, decomposition temperature = 315–395 °C), absolute ethanol, and methanol ≥99.8% were manufactured by Sigma-Aldrich, United Kingdom. Dimethyl sulfoxide (DMSO) was purchased from Honeywell. 5-Aminosalicyclic acid (5-ASA, 153.14 g/mol, decomposition temperature = 280 °C) purchased from FLUKA was donated by University College London School of Pharmacy. Polyvinyl alcohol (PVA) filament, 1.75 mm diameter, was purchased from RS-Pro. Phosphate buffer (pH 6.8) tablets were purchased from Millipore Corporation.

### 2.2. Methods

#### 2.2.1. Filament Preparation

Drug-containing filaments were prepared using the method described by Goyanes et al. [29,30]. In brief, 5 m of PVA filament was soaked in the drug-solvent mixture (100 mL) and stirred magnetically at 470 rpm for 24 h. The drug-loaded filaments were then heated (60 °C) in an oven (Pickstone Ovens, Island Scientific, Isle of Wight, UK) to facilitate rapid solvent evaporation (≈2 h). Resultant filaments were protected from light and moisture with aluminum foil and desiccants, respectively. Tagami et al. [31] suggested that this method of drug load requires the use of a saturated drug solution. Therefore, the drug concentrations chosen were based on drug concentrations (of FS and 5-ASA) used in previous studies [29,30]. Table 1 outlines the solvents, drug choices, and concentrations for the preparation of drug-loaded and solvent-soaked filaments. The names of the filament samples are included in brackets.

#### 2.2.2. Solid State Characterization of Filaments

##### X-Ray Powder Diffration

Structural characterization of filaments produced was conducted using a D/Max-BR diffractometer (RigaKu, Tokyo, Japan) with Cu Kα radiation operating at 40 kV and 15 mA (Cu Kalpha radiation) over the 2θ range 10−50° with a step size of 0.02° at 2°/min.

#### 2.2.3. 3D-Printed Drug Product Design and Optimization

Tablets were designed using TinkerCAD and were then imported as stl. format into MakerBot Desktop Beta (V3.10.1.1389) (MakerBot Industries. Brooklyn, NY, USA). Tablets were printed with PVA filament and drug loaded filaments using a MakerBot Replicator 2X (MakerBot Inc., Brooklyn, NY USA) with the following dimensions 10.45 × 10.54 × 1.2 mm [30]. Printer settings were standard resolution, 230 °C extrusion and 20 °C platform temperature, 100% hexagonal infill with raft option deactivated when printing drug-loaded tablets but activated for blank PVA tablets [24]. Printed tablets were assessed for weight uniformity.

#### 2.2.4. Morphology Studies

##### Scanning Electron Microscopy

Hitachi S5000 Emission Gun (FEG) (Hitachi, Maidenhead, UK) with Tungsten Tip (25 kV) was used to examine gold-coated (10 nm thickness) PVA tablet. Images were captured using secondary electron detector from ×70 to ×10.9 K magnification.

#### 2.2.5. Crushing Strength

**Figure 1:**
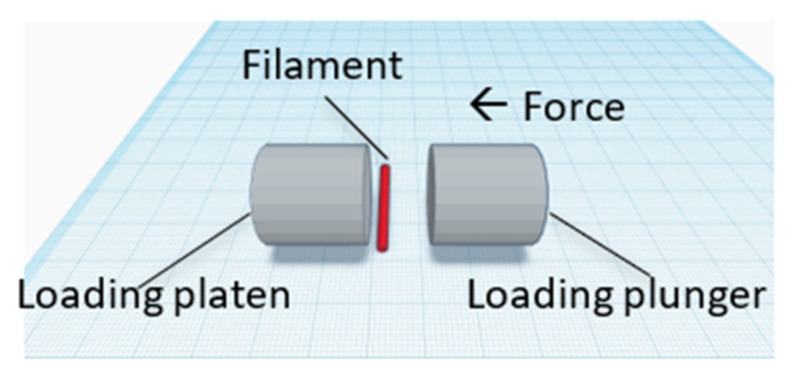
Orientation of filaments between loading plunger and platen. Increasing force was applied by loading plunger towards the platen. Direction of force is indicated by the arrow (←).

The crushing strength tests were conducted using a C50 Tablet Hardness and Compression tester (Engineering System, Nottingham, United Kingdom) on PVA and drug-loaded filaments. Figure 1 shows the sample orientation in the tester. Filament hardness was recorded as mean crushing strength (kg).

#### 2.2.6. Solubility, Drug Content, and In Vitro Drug Dissolution Studies

##### Solubility Studies

Solubility studies were conducted on FS and 5-ASA dissolved in the in vitro dissolution media, pH 6.8 phosphate buffer, and PVA solutions. Different amounts of PVA filament were dissolved in pH 6.8 phosphate buffers to prepare PVA-pH 6.8 solutions. Excess API (either FS or 5-ASA) was added to PVA solutions and vigorously stirred for 72 h at 37 ± 0.5 °C at 150 rpm. The saturated solutions were then filtered using a 0.45 μm membrane, and the API concentration in the filtrate was determined spectrophotometrically at 330 nm for 5-ASA and 490 nm for FS.

##### Calculation of Drug Content in PVA Filaments

Drug-loaded filaments were dissolved in pH 6.8 phosphate buffer and assayed spectrophotometrically (Perkin Elmer Lambda 35 Spectrophotometer) (PerkinElmer, Inc. Waltham, MA, USA) at 330 nm for 5-ASA and 490 nm for FS. PVA did not interfere with the UV analysis. Drug content was calculated using Equation (1) below.

(1) $$\mathrm{Drug}-\mathrm{content}\;\left(\%\;w/w\right)=\left(\frac{\mathrm{Weight}\;\mathrm{of}\;\mathrm{drug}\;\left(g\right)}{\mathrm{Weight}\;\mathrm{of}\;\mathrm{filament}\;\left(g\right)}\right)\times 100$$

##### In Vitro Drug Dissolution Studies

In vitro dissolution studies were conducted in pH 6.8 phosphate buffer (small intestine) at 37 ± 0.5 °C and a rotational speed of 100 rpm under non-sink conditions to observe any supersaturation effect from the solid dispersion products generated. At predetermined intervals, samples were withdrawn and filtered through a 0.45 μm filter and the filtrate was analysed spectrophotometrically at 330 nm for 5-ASA and 490 nm for FS.

#### 2.2.7. Statistical Analysis

Unpaired two-tailed *t*-test was performed using SigmaPlot V14.0 (Systat Software Inc. Berkshire, UK) with 95% significance level. *p* < 0.05 was regarded as significant.

## 3. Results

### 3.1. Solvent Choice Optimization

Solvents of increasing polarity: DMSO < EtOH < MeOH were investigated for their drug-loading efficiency into PVA filaments. Model drugs FS and 5-ASA are both polar; dissolving in solvents of higher polarity allows for generation of a larger concentration gradient for drug diffusion into the polymer (PVA) filament, therefore giving higher drug-loading in the presence of solvents of a higher polarity [32,33]. 

Table 2 shows mean drug content of the drug-impregnated filaments. DMSO completely solubilized PVA; therefore, the results below are based only on EtOH and MeOH. Coefficient of variation (%CV) ranged from 0.2% to 13.5%, highlighting the issue of non-uniform drug loading with soaking method, requiring further soaking apparatus choice selection.

Comparing single-drug-loaded filaments, MeOH was found to significantly increase drug loading compared to EtOH, irrespective of drugs (*p* < 0.05), fitting the hypothesis. The ratio of FS drug loading between MeOH and EtOH was approximately 4:1, whereas the ratio was smaller but statistically significant for 5-ASA (approximately 1.5:1 between MeOH and EtOH). Despite using the same drug concentration, 5-ASA content in FDC-MeOH filament was significantly higher (≈17 fold) than 5-ASA content in 5-ASA-MeOH filament (*p* < 0.01). MeOH was chosen to prepare FDC filament for the rest of the study due to its improved drug loading.

### 3.2. Filament Characterization

#### 3.2.1. Filament Hardness

It is expected that filament mechanical properties, hardness in particular, will change after drug impregnation, especially as most of the solvents used have the potential to degrade PVA [23]. Changes to filament properties can influence printability [34]. The crushing strength is frequently used in the pharmaceutical industry to describe the resistance of tablets to the application of a compression load [35]. In this study, we used the crushing strength to provide an indication of the changes in filament strength after soaking in EtOH and MeOH. Interestingly, the PVA filament crushing strength (kg) of 46.13 ± 0.89 kg decreased when the PVA filament was soaked (without the presence of the APIs) in EtOH and MeOH to 10.25 ± 1.04 kg and 10.78 ± 0.48kg, respectively. For single-drug-loaded filament, both FS-EtOH and 5-ASA-EtOH filaments had crushing strengths of 14.93 ± 1.74 kg and 14.65 ± 0.81 kg, respectively, however, these values significantly increased (*p* < 0.01) for MeOH-loaded drug filaments, FS-MeOH (25.34 ± 1.52 kg), and 5-ASA-MeOH (22.78 ± 1.21 kg). The crushing strength of FDC-MeOH filament was 16.77 ± 1.12 kg, similar to API-EtOH filaments (*p* > 0.05).

#### 3.2.2. Solid State Characterization of Filaments

**Figure 2:**
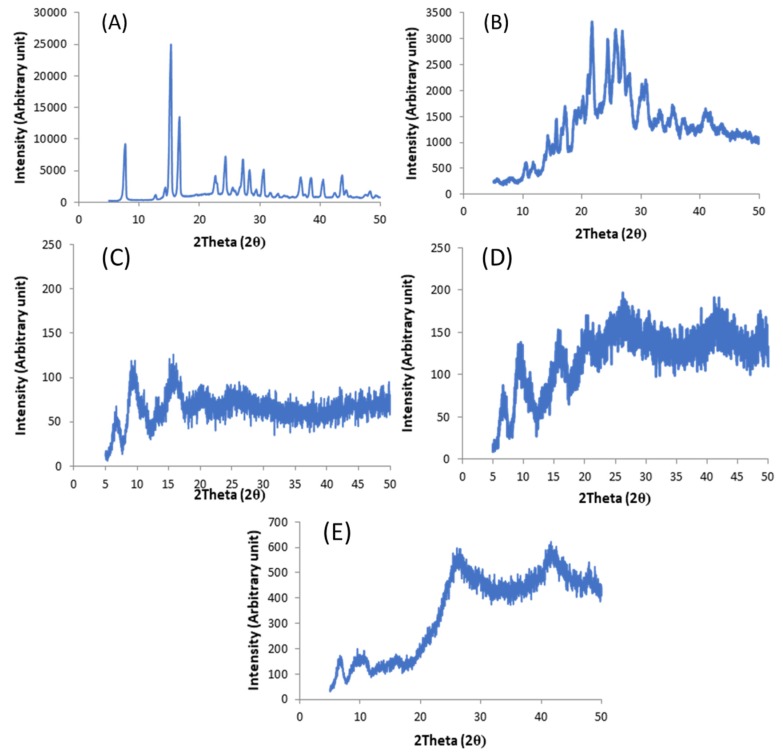
X-ray powder diffractograms of (**A**) 5-ASA, (**B**) FS, (**C**) blank polyvinyl alcohol (PVA) filament, (**D**) PVA-MeOH filament, and (**E**) FDC-MeOH filament data. Samples were scanned from 10–50° 2θ (stepwise: 0.02°, at 2°/min). Please note that different *y*-axis scales were used.

X-Ray Powder Diffraction (XRPD) studies were conducted to determine any potential physico-chemical changes to the API such as crystalline-amorphous transformation. XRPD diffractograms of raw materials of 5-ASA (Figure 2A) and FS (Figure 2B) both showed characteristic Bragg’s peaks, as confirmed by Groom et al. and Banic-Tomisic et al. [36,37]. XRPD studies on PVA filament, (Figure 2C) PVA-MeOH (Figure 2D), and FDC-MeOH filament (Figure 2E) showed characteristic amorphous halo with no evidence of crystalline API peaks.

### 3.3. Characterization of 3D-Printed Dosage Forms

#### Morphology Studies

**Figure 3:**
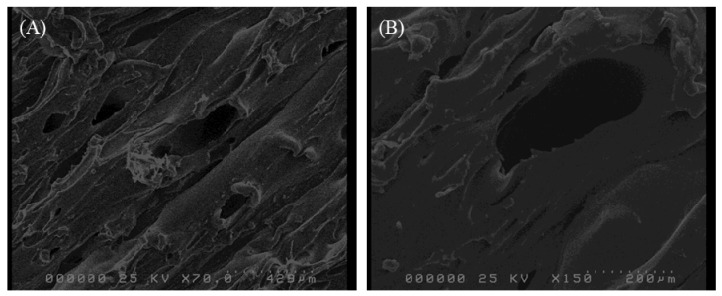
Surface morphology of PVA tablet (top view) at (**A**) ×70 and (**B**) ×150 magnification.

**Figure 4:**
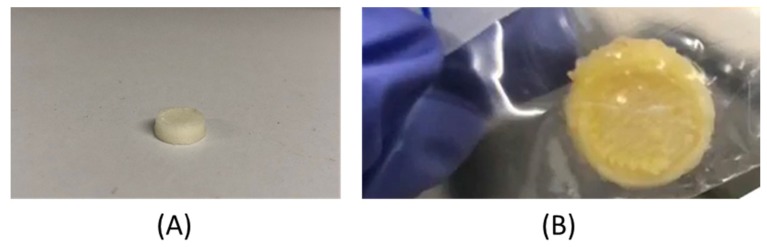
Representative image (**A**) printed PVA product and (**B**) three-dimensional (3D)-printed fixed-dose combination (FDC) drug product with dimensions 10.45 × 10.54 × 3.79 mm.

Layer-by-layer building of object via molten polymer fusion onto previously solidified extrudate layer during printing was expected to give extrudate-stacking appearance [38]. SEM images of a representative 3D printed dosage form is shown in Figure 3. Figure 3 clearly shows multiple voids on the dosage form surface, moreover, the dosage form obtained shows extrudate-stacking as expected and previously identified with FDM-printed dosage forms [25]. Representative images of printed products are provided in Figure 4.

### 3.4. In Vitro Dissolution

Saturated solubility of FS and 5-ASA was determined in in vitro dissolution medium at increasing PVA concentration. FS and 5-ASA saturated solubility in pH 6.8 phosphate buffer was 385.82 and 2.98 mg/mL, respectively. In the presence of increased PVA concentration (0.6% *w*/*v* and 0.4% *w*/*v*), FS and 5-ASA saturated solubility was 465.75 and 1.60 mg/mL, respectively.

**Figure 5:**
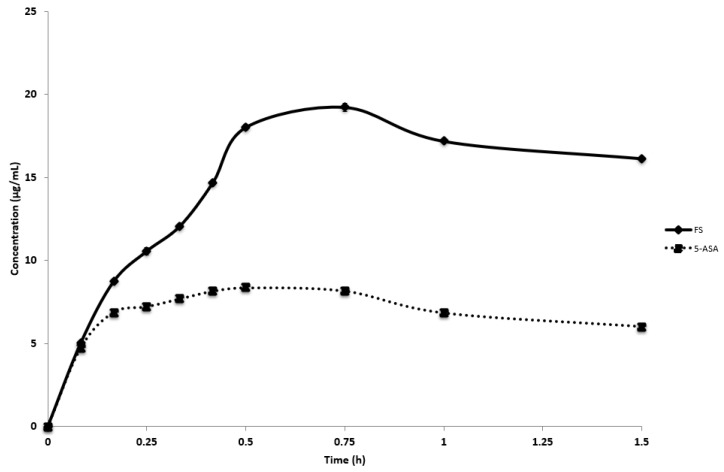
In vitro dissolution profile of 3D-printed FDC-MeOH dosage form in phosphate buffer pH 6.8. Error bars representing standard deviations.

FS and 5-ASA raw materials achieved complete dissolution within 5 min (data not shown); therefore, Figure 5 shows dissolution profiles of the 3D-printed FDC-MeOH product. Amorphous solid dispersion (ASD) formulations are known to experience rapid dissolution before recrystallizing at a rate corresponding to PVA concentration, acting as a crystallization inhibitor [39,40,41]. The 3D-printed FDC-MeOH product dissolution showed characteristic ASD spring-and-parachute dissolution profile of both FS and 5-ASA. Peak concentration was achieved at *t* = 30 min for 5-ASA (8.36 ± 0.06 µg/mL), corresponding to a dissolution rate of 16.71 µg/mL/h and *t* = 45 min for FS (19.24 ± 0.24 µg/mL).

## 4. Discussion

The main aim of this study was to investigate the effect of different solvent in loading drugs on printable PVA filament. The impregnation method used has already been used in studies by Goyanes et al. where drug contents for 5-ASA- and FS-loaded PVA filaments were 0.063 ± 0.001% *w*/*w* and 0.29 ± 0.01% *w*/*w*, respectively [29,30]. In our study, we were able to achieve approximately three-fold and one-fold higher FS and 5-ASA drug-loading, respectively, compared to the studies of Goyanes et al. In particular, it was noted that MeOH-loaded drug filaments had significant improvement in drug-loading of polar FS and 5-ASA compared to EtOH, highlighting the importance of matching solvent drug polarity when using the drug impregnation method. Apart from solvent polarity, drug dielectric constant, solubility, as well as temperature and hygroscopicity can affect drug loading [42]. The presence of FS may have altered MeOH dielectric constant, facilitating H^+^ ion dissociation from 5-ASA, and increasing 5-ASA solubility in MeOH; this proposed synergistic effect requires further investigation [43,44,45,46]. 

Our findings on changes in filament hardness before and after drug impregnation showed that drug and solvent molecules can interpose between polymer chains, weakening polymer–polymer interaction and increasing chains movements, resulting in reducing PVA filament hardness by more than 50% after drug-loading and solvent-soaking [47,48,49]. Because plasticizing effect has previously been found to increase with plasticizer concentration, FDC-MeOH filament, having significantly higher drug loading compared to single-drug filaments, resulted in lower hardness compared to other MeOH-loaded filaments.

The drug dissolution profile of the FDM-printed monolithic FDC tablet (FS and 5-ASA) was evaluated. The advantage of this drug product as a solid dispersion is based on the water-soluble matrix that provides activation energy to drive crystallization [28,50,51,52,53]. As drug release from PVA is also regulated by polymer dissolution, all printed tablets in the current study also exhibited spring-and-parachute profile with crystallization inhibited depending on PVA concentration.

The reason for rapid supersaturation resulting in higher maximum concentration could be that the system had insufficient time to induce crystallization when transformation from stable to metastable supersaturation state was quick [54,55]. In the current study, 5-ASA released from the the 3D-printed drug product had initial rapid de-supersaturation; however, the rate declined alongside with the reduction of 5-ASA in the system. This could be because polymer has a greater precipitation inhibitory effect at lower supersaturation [56,57,58]. 

## 5. Conclusions

The use of FDM 3DP technology in the pharmaceutical industry is hindered by several formulation challenges, which the current study aimed to address. This study investigated the solvent influence on optimal drug filament impregnation with an identification that MeOH possessed superior properties compared to EtOH for FS and 5-ASA. Using this method and solvent choice, reasonable drug loading of both FS and 5-ASA onto a single PVA filament was achieved. A 3D-printed solid dispersion FDC drug product was successfully designed and characterized with favourable release profiles and behaviours. Further studies using clinically relevant drugs would be advantageous for the advancement of the work in this area.

## Figures and Tables

**Table 1:** Solvent, drug, and drug concentrations used.

Active Pharmaceutical Ingredient (API)	Ethanol (EtOH)	Methanol (MeOH)	Dimethyl Sulfoxide (DMSO)
	PVA		
Fluorescein sodium (FS)	2.0% *w*/*v* (FS-EtOH)	2.5% *w*/*v* (2.5%FS-MeOH)	2.5% *w*/*v* (FS-DMSO)
5-aminosalicyclic acid (5-ASA)	1.0% *w*/*v* (5-ASA-EtOH)	1.25% *w*/*v* (5-ASA-MeOH)	1.25% *w*/*v* (5-ASA-DMSO)
FS and 5-ASA		2.5% *w*/*v* FS and 1.25% *w*/*v* 5-ASA (FDC-MeOH)	

**Table 2:** Mean drug-content (% *w*/*w*) of drug-loaded filaments prepared via drug impregnation method.

Solvents	Drug-Loaded Filaments	Drug Loading (% *w*/*w*)
Ethanol	FS-EtOH	1.19 ± 0.161
	5-ASA-EtOH	0.10 ± 0.001
Methanol	FS-MeOH	4.89 ± 0.449
	5-ASA-MeOH	0.17 ± 0.007
	FDC-MeOH	FS: 6.16 ± 0.197 5-ASA: 2.97 ± 0.362
